# Global Perspectives on Arbovirus Outbreaks: A 2020 Snapshot

**DOI:** 10.3390/tropicalmed5030142

**Published:** 2020-09-07

**Authors:** Rebekah C. Kading, Aaron C. Brault, J. David Beckham

**Affiliations:** 1 Department of Microbiology, Immunology, and Pathology, Colorado State University, Campus Delivery 1690, Fort Collins, CO 80523, USA; 2 Division of Vector-borne Diseases, Arboviral Diseases Branch, Centers for Disease Control and Prevention, Fort Collins, CO 80521, USA; zlu5@cdc.gov; 3 Departments of Medicine, Neurology, and Immunology & Microbiology, Division of Infectious Diseases, University of Colorado School of Medicine, Rocky Mountain Regional VAMC, 12,700 E. 19th Avenue, B168, Denver, CO 80045, USA; david.beckham@cuanschutz.edu

**Keywords:** mosquito, tick, emerging infectious diseases, one health, vector-borne diseases

When this special issue was first conceived in early 2019, we never anticipated that the publication of this collection of articles would be happening during a pandemic. While this outbreak collection is focused on viruses transmitted by arthropods, its release concurrent with the SARS-CoV-2 pandemic and international health emergency provides an appropriate context in which to draw attention to research focused on other high-consequence, epidemic-causing viruses that may be next to emerge on the global stage.

Arthropod-borne pathogens account more than 17% of infectious diseases, affect millions of people around the world each year, and comprise a significant proportion of emerging human pathogens [1,2,3,4]. Dengue, as the most widespread arboviral disease, causes more than 90 million cases and approximately 40,000 deaths per year [3]. The emergence and explosive spread of Zika virus (ZIKV) has recently challenged the global health infrastructure to diagnose and differentiate ZIKV infections from infections with closely related arboviruses and to minimize the catastrophic effects of congenital infection. Additional arboviruses including Rift valley fever, Mayaro, West Nile, chikungunya, and tick-borne encephalitis viruses have captured the attention of the scientific community as emerging public health threats [3,5]. As the world responds to outbreak after outbreak, research on: surveillance and preparedness, virus transmission dynamics, ecology and epidemiology, diagnosis and treatment, public attitudes and practices and existing barriers and challenges to outbreak prevention and control of these and other emerging viruses will be needed to contribute to more effective mitigation strategies and for protecting public health.

This Special Issue comprises a highly diverse group of articles from around the world, which highlight various aspects of arbovirus outbreaks in endemic regions and in areas of introduction. This issue showcases the important work that is being done to mitigate epidemic activity and protect human and animal health from emerging arboviral threats, as well as provides a unique insight into past epidemics. Articles in this issue include coverage of viruses transmitted by ticks as well as mosquitoes, discussions of clinical, epidemiological, and entomological perspectives, and includes emerging arboviruses from every hemisphere. Below is a brief tour of the articles in this collection (Figure 1).

*Southeast Asia*: The two studies included in this collection from Southeast Asia provide corresponding perspectives on the transfer or “spillover” of zoonotic pathogens from natural transmission cycles into the human population. Young et al. [6] described the blood feeding patterns of mosquitoes in Malaysian Borneo, in an area undergoing significant changes in land cover and land use. They provided novel insights into how the host utilization patterns of some major mosquito vectors in different land use types could influence the spread and spillover of arboviruses, including Japanese encephalitis virus, between natural and epidemic cycles. As a complement to this work, Ab Hamid et al. [7] studied the vertical stratification of *Aedes* mosquitoes responsible for the transmission of dengue virus (DENV) in high-rise buildings in Malaysian cities. They reported mosquito infestations up to 21 stories high, demonstrating a unique risk of arbovirus transmission in urban settings [7].

*South America*: While screening sera from human patients in Sinop city, Brazil, de Silva Pessosa Vierra et al. [8] documented concurrent circulation of both Mayaro (MAYV) and chikungunya (CHIKV) viruses. While the MAYV genomes detected matched other strains known to be present, the CHIKV genome detected from one patient was of the East/Central/South African (ECSA) genotype. This genotype was distinct from strains already circulating in this area and represented an important discovery, that there was a separate introduction event of CHIKV into Brazil [8]. The simultaneous circulation of *Aedes* mosquito-borne viruses including DENV, CHIKV, ZIKV, and MAYV, and the stress this poses to the human population and health infrastructure in Brazil is discussed in detail by Magalhaes et al. [9].

*Africa*: This collection covers two hemorrhagic fever viruses endemic to Africa, Rift Valley fever virus (RVFV) and Crimean Congo hemorrhagic fever virus (CCHFV), both of which are listed as “select agents” [10] due to the severity of disease they could cause, as well as potential use as agents of bioterrorism. Rift Valley fever virus is endemic to Africa where it causes large epizootics typically associated with climate patterns and rainfall, and causes significant morbidity and mortality in both humans and animals [11]. This virus has spread to Saudi Arabia, Madagascar, and other island nations in the Indian Ocean (i.e., Mayotte, Comoros), but has not yet emerged in a transoceanic location. Mitigation of a RVFV outbreak carries with it many unique considerations, and would involve mobilization of diverse agencies focused on public health, animals and agriculture, and biosecurity. To this end, Grossi-Soyster and LaBeaud [12] reviewed major considerations for such risk mitigation pertaining to future outbreaks of RVFV, with a special emphasis on vaccines, travelers and tourism. Crimean Congo hemorrhagic fever virus, transmitted by ticks, was originally described from both central Africa as well as the Crimean region of Russia. This virus has alarmingly sustained continual epizootic activity from Africa north through Western Asia in recent years. Sorvillo et al. [13] comprehensively reviewed and discussed a One Health approach to CCHFV prevention and included a special focus on knowledge gaps that are critically important to address.

*North America*: The United States has experienced invasions of West Nile and Zika viruses in recent decades, with continual threats of endemic re-emerging mosquito-borne viruses such as St. Louis, LaCrosse, Powassan, and Eastern equine encephalitis viruses. In 1971, a transboundary outbreak of Venezuelan equine encephalitis (VEE) virus epidemic strain 1b invaded South Texas. In a letter to the editor, McLean [14] provided a valuable first-hand account of the interagency response to this outbreak, and how this outbreak response remains the sole example of the successful prevention of VEE establishment in the United States. To build on this historical perspective, Kading et al. [15] provided a 30-year analysis on the reactionary response of funding agencies to the emergence and invasion of mosquito-borne viruses in the Americas and how these events have also stimulated the innovative development of traps and augmented surveillance capacity. As the most recent arbovirus introduction to the United States, ZIKV infections have been mostly associated with travelers, however local transmission was documented in areas of Florida and Texas [16,17]. To this end, Hinojosa et al. [16] reported locally-acquired cases of ZIKV in South Texas, particularly as surveillance efforts were increased out of specific concerns of virus infections in pregnant women.

*Europe*. Tick-borne encephalitis (TBE) is a severe infection of the central nervous system, caused by tick-borne encephalitis virus (TBEV) in the family *Flaviviridae*. Incidence of TBE has increased in recent years throughout Europe, spread to new endemic foci, and became a notifiable disease in the European Union in 2012 [18,19]. The TBE vaccine licensed in Europe is recommended for all age groups in endemic areas with incidence rates exceeding 5 per 100,000 [19,20], however vaccination rates are very low [19]. Riccò et al. [18] conducted a knowledge, attitudes and practices survey of tick-borne encephalitis among occupational physicians in Italy, and found a lack of knowledge of TBE and low vaccine literacy among this stakeholder group. Improving knowledge of TBE, behavioral practices that prevent tick-bites, and vaccination would help prevent the spread of tick-borne infections such as TBE in Italy and elsewhere [18]. West Nile virus (WNV) has been circulating throughout Europe since the 1990s. A dramatic increase in WNV infections in multiple countries in Southern Europe was observed during 2018 in both humans and horses [21]. Castaldo et al. [22] provided a case report on two human patients from Italy. Clinical disease was characterized by atypical neurological presentation in these patients diagnosed with WNV neuroinvasive disease, involving the brainstem. The authors encouraged providers to remain alert to unusual disease presentations involving the central nervous system [22].

## Figures and Tables

**Figure 1:**
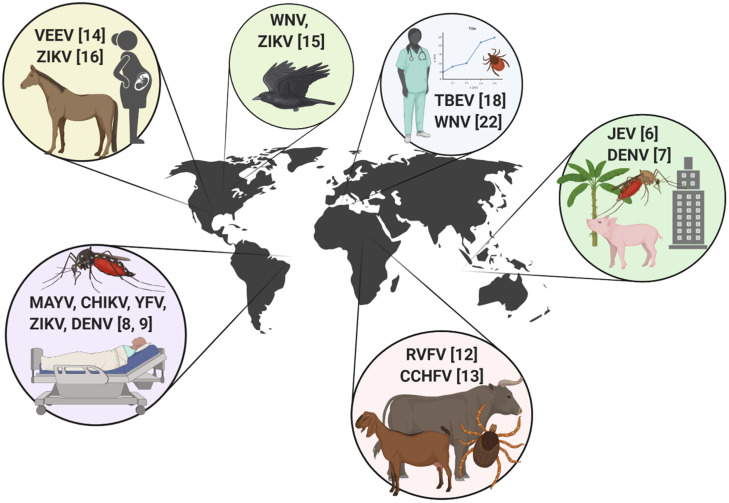
International scope of arbovirus outbreak-related articles included in the “Arthropod-borne Viruses: The Outbreak Edition” special collection. Venezuelan equine encephalitis virus (VEEV), Zika virus (ZIKV), West Nile virus (WNV), tick-borne encephalitis virus (TBEV), Japanese encephalitis virus (JEV), dengue virus (DENV), Rift Valley fever virus (RVFV), Crimean-Congo hemorrhagic fever virus (CCHV), Mayaro virus (MAYV), chikungunya virus (CHIKV), and yellow fever virus (YFV). Numbers indicate the associated reference.
